# Expanded-access use of elamipretide in a newborn with Barth syndrome: a case report

**DOI:** 10.1093/ehjcr/ytaf030

**Published:** 2025-01-22

**Authors:** Laura Ortmann, Danita Velasco, Jason Cole

**Affiliations:** Department of Pediatrics, University of Nebraska Medical Center, Children’s Nebraska, 8200 Dodge St, Omaha, NE 68114, USA; Department of Pediatrics, University of Nebraska Medical Center, Children’s Nebraska, 8200 Dodge St, Omaha, NE 68114, USA; Department of Pediatrics, University of Nebraska Medical Center, Children’s Nebraska, 8200 Dodge St, Omaha, NE 68114, USA

**Keywords:** Barth syndrome, Elamipretide, Cardiomyopathy, Mitochondria, Cardiolipin, Case report

## Abstract

**Background:**

Barth syndrome (BTHS) is a rare genetic disease, with no approved curative therapies, characterized by abnormally developed cardiolipin, resulting in mitochondrial dysfunction. Cardiomyopathy, a common clinical manifestation of BTHS, often appears in infancy. Elamipretide, an investigational drug that binds to cardiolipin on the inner mitochondrial membrane, leads to improved membrane stability, enhanced adenosine triphosphate production, and reduced reactive oxygen species. This patient case aims to further support elamipretide’s role in treating BTHS infants.

**Case summary:**

We present an infant diagnosed *in utero* with BTHS who demonstrated a moderately dilated left ventricle (LV) with an LV ejection fraction (LVEF) of 20% at birth. He was transferred to a tertiary children’s hospital where he was intubated and administered medications for haemodynamic support. After several weeks, the patient was extubated and his LVEF improved, although still below normal. On day of life (DOL) 34, therapy with daily IV elamipretide (0.25 mg/kg increased to 0.5 mg/kg on DOL39) began, followed by standard-of-care oral heart failure medications. Subsequent echocardiograms demonstrated improvement of LVEF to near-normal levels. He was weaned off oxygen completely on DOL49 and discharged home on DOL61 on daily subcutaneous elamipretide 0.5 mg/kg and oral heart failure medications. His most recent echocardiogram showed improvement of LVEF to 60%.

**Discussion:**

Our case suggests that elamipretide may have contributed to the improvement of LV function in this BTHS infant, supporting elamipretide’s early use in BTHS. Our findings align with the previous studies in which elamipretide treatment demonstrated normalization of mitochondrial function and improvement in LV function.

**Learning points**

- Barth syndrome (BTHS) is a rare, genetic disorder, with no approved curative treatment, primarily presenting with cardiomyopathy due to underlying abnormal cardiolipin and subsequent mitochondrial dysfunction.
- Elamipretide is a mitochondria-targeting agent that binds to cardiolipin and has shown promise in BTHS patients in clinical trials through improvements in functional assessments and cardiac function.
- Our case report suggests that elamipretide therapy may have contributed to the maintenance of LV function in an infant with cardiomyopathy in the setting of BTHS.

## Introduction

Barth syndrome (BTHS) is a rare, X-linked genetic disease estimated to occur in 1 in 1 million male live births.^1^ Barth syndrome pathophysiology involves abnormal cardiolipin, an inner mitochondrial membrane phospholipid critical to mitochondrial function, that occurs due to mutations in the *TAFAZZIN* gene encoding an acyltransferase necessary for the remodelling and maturation of cardiolipin.^2^ Cardiomyopathy, the most common clinical manifestation of BTHS, occurs in ∼90% of patients (70% diagnosed in infancy/12% requiring cardiac transplantation at a median age of 1.7 years).^3,4^

No approved therapies exist for BTHS; however, the US Food and Drug Administration has provided guidance on expanded access for using investigational drugs, such as elamipretide, for patients with serious diseases lacking satisfactory therapeutic alternatives.^5^ Elamipretide is a mitochondria-targeting peptide that binds to cardiolipin, leading to improved membrane stability, enhanced adenosine triphosphate (ATP) production, and reduced production of reactive oxygen species (ROS).^6,7^ Elamipretide was assessed for the treatment of BTHS patients in the TAZPOWER trial and the subsequent open label extension (OLE), demonstrating functional and cardiac function improvements in elamipretide-treated patients.^8,9^ Here, we describe elamipretide use under the Expanded Access Program (EAP) in a BTHS newborn with the objective of providing further support for elamipretide’s therapeutic role in BTHS infants.

## Summary figure

**Figure:**
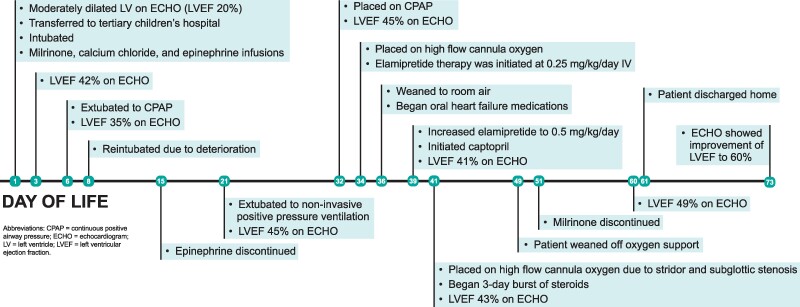


## Case presentation

Prior to the patient’s birth, the mother was closely followed during pregnancy, having been a confirmed carrier of BTHS. The mother’s family history consisted of the following: maternal half-brother who died at 1.5 years of age from cardiomyopathy (no genetic testing); maternal grandfather with neutropenia and cardiomyopathy; and half first cousin with genetically confirmed BTHS who underwent heart transplantation. During her pregnancy, a BTHS was confirmed through amniocentesis, identifying the pathogenic familial variant in *TAFAZZIN* (c.589G>A; p.Gly197Arg).

The 20-week ultrasound was normal for anatomic structures in the foetus. Paediatric cardiology followed the mother prenatally, with care including regular foetal echocardiograms. Foetal echocardiograms at 28- and 35-week gestation demonstrated biventricular dilation, mild to moderate right ventricular (RV) dysfunction, and normal left ventricular (LV) function. Stable foetal echocardiograms supported a planned, local hospital delivery.

After an uneventful birth, the first echocardiogram showed a moderately dilated LV with normal wall thickness and a normal-sized RV with severely depressed biventricular function [LV ejection fraction (LVEF) ∼20%] (Echocardiogram image available in Supplementary material, *Video S1*). Due to the echocardiogram findings, the patient transferred to our local children’s hospital for further management. After admission, he was intubated due to rising lactates (peak 15 mmol/L) and administered milrinone, calcium chloride, and epinephrine infusions for haemodynamic support on day of life (DOL) 1. The patient was subsequently weaned off the calcium infusion but maintained on milrinone and low-dose epinephrine.

While working with insurance to consider transferring the patient for ventricular assist device placement and heart transplantation evaluation, the patient was extubated to continuous positive airway pressure (CPAP) on DOL6. However, he experienced elevated lactates, respiratory distress, tachycardia, worsened kidney function, and decreased near infrared spectroscopy, and thus, he was reintubated on DOL8. The epinephrine dose was titrated down and discontinued on DOL15.

On DOL21, the patient was extubated to non-invasive positive pressure ventilation with a slow wean to CPAP (DOL32) and transition to heated high-flow cannula (DOL34). The patient was enrolled into the EAP (Stealth BioTherapeutics, Needham, MA) for elamipretide, consent was obtained from the patient’s guardian, and drug administration was approved by the local Institutional Review Board. On DOL34, intravenous elamipretide therapy was initiated at 0.25 mg/kg/day and increased to 0.5 mg/kg/day on DOL39. The patient was weaned to room air on DOL36, but developed stridor and was found to have grade II subglottic stenosis; therefore, he was placed back on high-flow cannula (DOL41) and administered a 3-day steroid burst. On DOL36, the patient began oral heart failure medications, including carvedilol 0.05 mg/kg and spironolactone 1.5 mg/kg. Captopril was added to his regimen at 0.05 mg/kg three times daily on DOL39. Intermittent furosemide doses were administered as needed. The patient weaned off oxygen on DOL49. All oral heart failure medications were titrated up appropriately over time while the dose of milrinone was titrated down, completely discontinuing the infusion on DOL51. During this time, the patient also began full volume feeds via nasogastric tube.

Throughout his hospital course, the patient’s echocardiograms improved with normalization of right-sided function and improved LV function to the low-normal to mildly depressed range. During this time, he made steady progress and was able to demonstrate weight gain on all oral feeds. The patient was discharged home on DOL61 on subcutaneous elamipretide 0.5 mg/kg/day, captopril 0.3 mg/kg, carvedilol 0.3 mg/kg, spironolactone 1.5 mg/kg, and furosemide 3 mg. After discharge, he continued to maintain oral feeds and was reported to be doing well by his mother (Echocardiogram image from DOL 73 available in Supplementary material, *Video S2*). His most recent echocardiogram (DOL88) showed LVEF improvement to 60% (from 49% at time of discharge). During hospitalization and outpatient follow-up (about 1-month post-discharge at the time of submission), the mother has reported no side effects from elamipretide. We plan to maintain the patient on weight-based dosing of elamipretide and monitor with regular echocardiograms.

## Discussion

**Figure 1:**
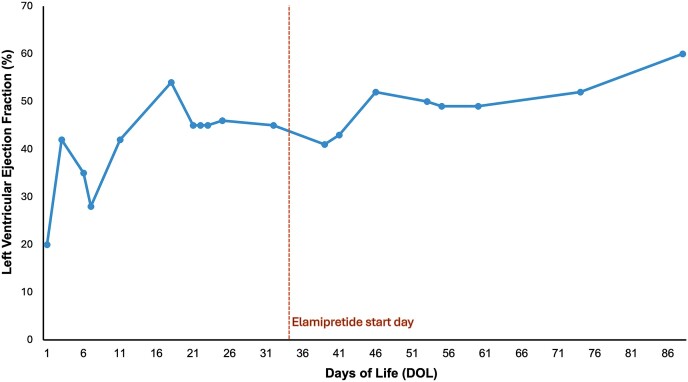
Left ventricular ejection fraction over the patient’s early life.

This case describes the expanded-access use of elamipretide in an acutely ill newborn with BTHS and supports findings of previous studies and case reports.^8,10^ While injection site reactions are a well-known adverse event of subcutaneous elamipretide, this patient did not appear to experience any side effects, aligning with the favourable safety profile of elamipretide reported in other studies/cases.^8–10^ Our patient showed remarkable improvement in cardiac function as evidenced by his echocardiographic findings (*Figure 1*), which significantly contributed to his overall recovery. Given his impressive improvement, the patient was never placed on the heart transplant list and did not require mechanical circulatory support.

**Figure 2:**
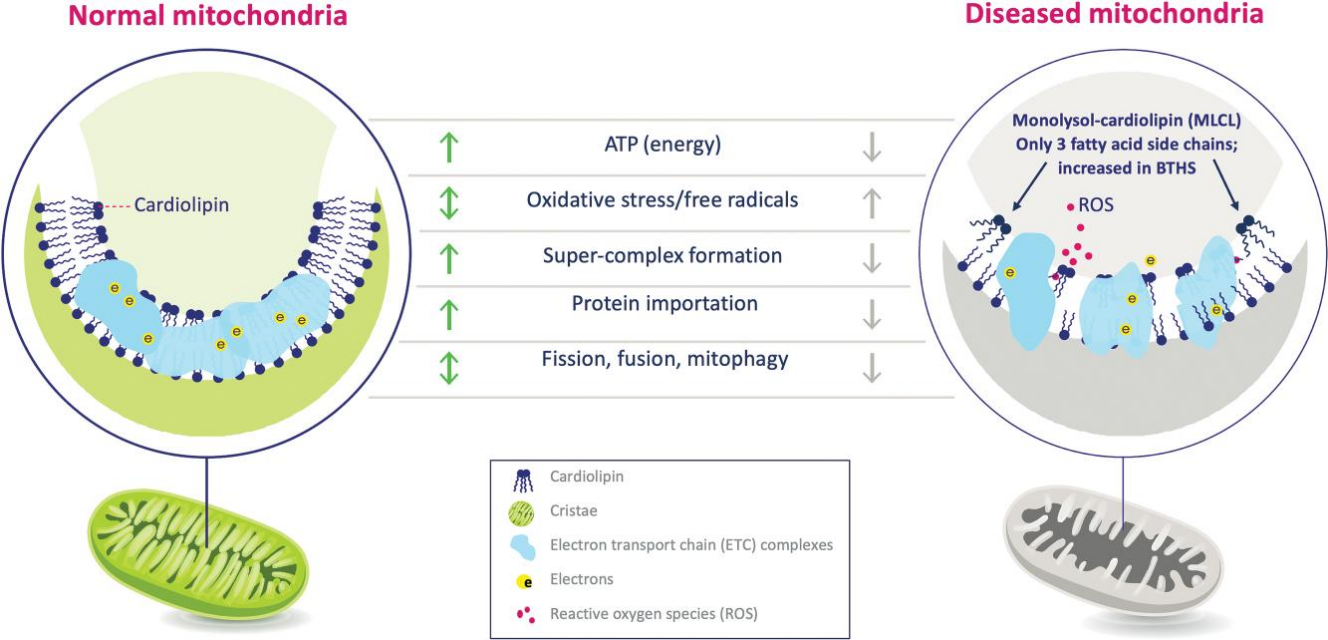
Proposed disease pathway of Barth syndrome. ATP, adenosine triphosphate; ROS, reactive oxygen species.

**Figure 3:**
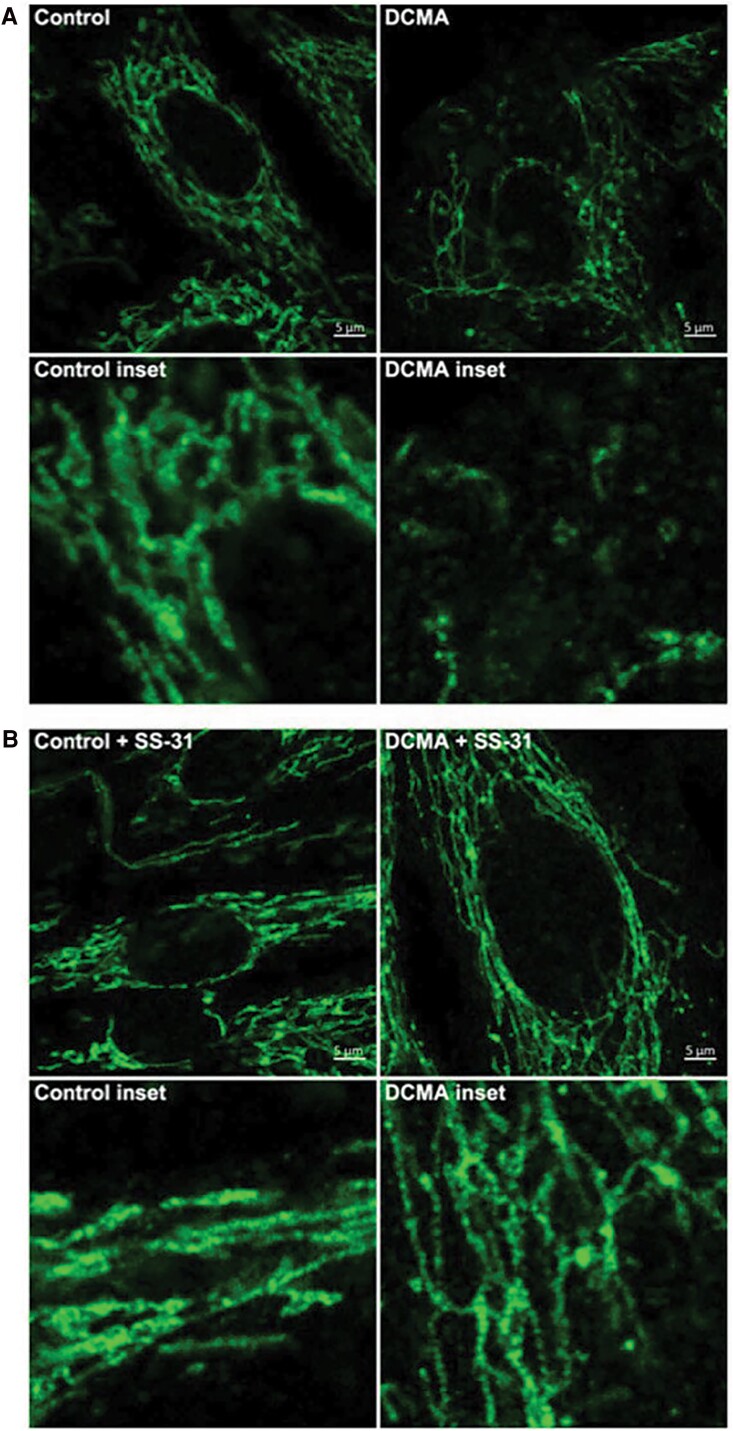
Fluorescence imaging of mitochondria from control and dilated cardiomyopathy patient-derived fibroblasts without treatment (*A*) and with elamipretide (SS-31) treatment showing less fragmentation (*B*). Machiraju *et al*. 2019; *Frontiers in Cardiovascular Medicine*.

**Figure 4:**
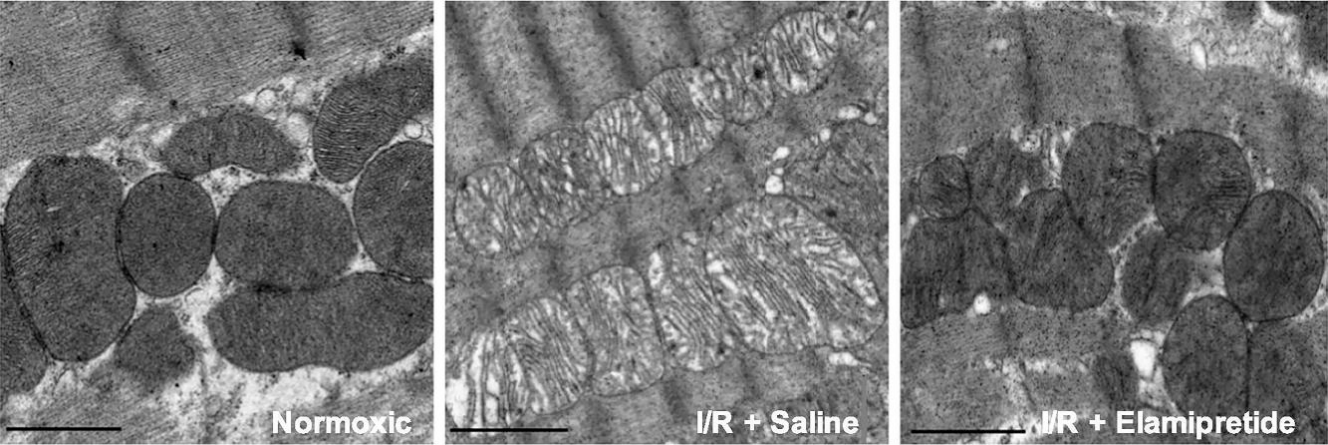
Electron microscopy images of heart cristae from rats exposed to ischaemia/reperfusion, demonstrating improvements with elamipretide treatment. Allen *et al*. 2020; *Communications Biology*.

Elamipretide is a logical choice as a therapeutic agent for BTHS patients as the interaction between elamipretide and cardiolipin species is not dependent on the content or composition of the cardiolipin side chains; therefore, elamipretide can interact with monolysocardiolipin (MLCL) in a similar ratio.^11^ Barth syndrome causes mitochondrial dysfunction through the reduction in mature cardiolipin and accumulation of MLCL (*Figure 2*).^12^ In previous preclinical/animal studies, elamipretide therapy demonstrated normalization of mitochondrial structure (*Figures 3* and *4*) and function through an improved rate of ATP synthesis and reduced ROS formation.^7,13^

In animal models, elamipretide increased LVEF, reduced ventricular hypertrophy, and improved LV systolic and diastolic function.^13,14^ Furthermore, daily elamipretide therapy significantly improved the LV stroke volume, a key component of cardiac output and LVEF, in BTHS patients treated in the phase 2/3 TAZPOWER trial and OLE.^9^ Data from the OLE demonstrated a marked reverse remodelling effect of the heart (greater than 40% improvement in LV volumes) in patients treated with elamipretide.^8,9^ This study was conducted in older patients; available data on the efficacy of elamipretide in newborns are limited. In a recent publication, an 11-month-old infant with BTHS was reported to have significant improvement in cardiac function upon elamipretide administration, as evidenced by echocardiogram, such that the patient was removed from LVAD support.^10^

The findings in our case report suggest that, while the rescue medications and oral heart failure therapies likely contributed to improvement and maintenance in cardiac function, respectively, as has been published to occur in some BTHS patients,^15^ elamipretide therapy may have contributed to the maintenance of LV function by increasing the maximal rate of ATP synthesis and normalizing mitochondrial function. This provides promise for elamipretide as an effective targeted therapy for BTHS patients when started in infancy.

## Supplementary Material

ytaf030_Supplementary_Data

## Data Availability

The data underlying this article will be shared on reasonable request to the corresponding author.

## Lead author biography

Dr Laura Ortmann is the Director of Cardiac Critical Care at Children’s Nebraska and associate professor at the University of Nebraska College of Medicine. Her clinical research interests include resuscitation, post-operative cardiac care, education, and fluid overload. Prior to her outstanding career, Dr Ortmann earned her medical degree from the University of Nebraska Medical Center and completed a residency in paediatrics at Children’s Mercy Hospital and fellowships in paediatric critical care medicine and cardiac critical care at the University of Arkansas for Medical Sciences and Lucile Packard Children’s Hospital, respectively.
