# Supplementary material for: Expanded-access use of elamipretide in a newborn with Barth syndrome: a case report
Source: Eur Heart J Case Rep. 2025 Jan 22;9(2):ytaf030. doi: 10.1093/ehjcr/ytaf030 (PMC11799937; doi:10.1093/ehjcr/ytaf030)
Supplement: ytaf030_Supplementary_Data [file ytaf030_supplementary_data.zip › Supplementary captions.docx]

**Supplemental File (Video) 1:** Four chamber view obtained on day of birth showing a moderately dilated left ventricle with severely depressed biventricular function.

**Supplemental File (Video) 2:** Four chamber view obtained at first outpatient visit (DOL73) showing improvement in left ventricular dilation and function. DOL = day of life
